# A KPI-Based Probabilistic Soft Sensor Development Approach that Maximizes the Coefficient of Determination

**DOI:** 10.3390/s18093058

**Published:** 2018-09-12

**Authors:** Yue Zhang, Xu Yang, Yuri A. W. Shardt, Jiarui Cui, Chaonan Tong

**Affiliations:** 1 Key Laboratory of Knowledge Automation for Industrial Processes of Ministry of Education, School of Automation and Electrical Engineering, University of Science and Technology Beijing, Beijing 100083, China; s20160638@xs.ustb.edu.cn (Y.Z.); cuijiarui@ustb.edu.cn (J.C.); tcn@ies.ustb.edu.cn (C.T.); 2 Department of Automation Engineering, Technical University of Ilmenau, 98684 Ilmenau, Thuringia, Germany; yuri.shardt@tu-ilmenau.de

**Keywords:** soft sensor, coefficient of determination maximization strategy, expectation maximization (EM) algorithm, Gaussian mixture model (GMM), alumina concentration

## Abstract

Advanced technology for process monitoring and fault diagnosis is widely used in complex industrial processes. An important issue that needs to be considered is the ability to monitor key performance indicators (KPIs), which often cannot be measured sufficiently quickly or accurately. This paper proposes a data-driven approach based on maximizing the coefficient of determination for probabilistic soft sensor development when data are missing. Firstly, the problem of missing data in the training sample set is solved using the expectation maximization (EM) algorithm. Then, by maximizing the coefficient of determination, a probability model between secondary variables and the KPIs is developed. Finally, a Gaussian mixture model (GMM) is used to estimate the joint probability distribution in the probabilistic soft sensor model, whose parameters are estimated using the EM algorithm. An experimental case study on the alumina concentration in the aluminum electrolysis industry is investigated to demonstrate the advantages and the performance of the proposed approach.

## 1. Introduction

With the increasing demands placed on industry, requiring a decrease in the defective rate of products, better economic efficiency, and improved safety, there has been a growing demand to develop and implement approaches that can improve the overall control strategy [1]. The first issue that needs to be solved is achieving accurate and real-time estimation of key performance indicators (KPIs) [2]. The difficulty is that these KPIs are usually not easy to measure, or the measurement has significant time delay. Even if some KPIs are measurable, due to the complexity and nonlinearity of modern industrial systems and their complex working conditions, the KPIs may be extremely unreliable [3]. One way to solve the above problems is to develop a soft sensor, which seeks to select a group of easier-to-measure secondary variables that are correlated with the required primary variables (i.e., KPIs in this paper), so that the system is capable of providing process information as often as necessary for control [4,5]. In the development of a successful soft sensor, a good process model is required. The process models can be divided into two major categories: first principles models and data-driven models [6,7]. Although it is desirable to apply mass and energy balances to build a complete first principles model, lack of process knowledge, plant–model mismatch, and nonlinear characteristics limit the applicability of such an approach to the simplest processes. As an alternative, data-driven soft sensors are developed from historical data without necessarily considering any outside process knowledge. Data-driven soft sensors, which solely use available process data to develop a model of the process, have recently attracted considerable attention and have been successfully applied in many fields [8], such as fault detection (FD) and process monitoring, that are important for many industrial processes. Serdio [9] introduced an improved fault detection approach based on residual signals extracted online from system models identified by high-dimensional measurements provided by the multisensor network. The data-driven system identification model can also be combined using multivariate orthogonal space transformations and vectorized time-series models to achieve enhanced residual-based fault detection in condition monitoring systems equipped with a multisensor network [10]. Shardt [11] proposed a data-driven design of a diagnostic-observer-based process monitoring method, which was extended to include the ability to detect changes given infrequent KPI measurements. Yan [12] and Gabrys [13] introduced the most popular data-driven soft sensor modelling techniques, as well as discussing some issues in soft sensor development and maintenance and their possible solutions. Data-driven methods can be divided into three categories: models based on statistical analysis, models based on statistical learning theory [14], and models based on artificial intelligence [15].

Of interest for this paper are models developed using statistical methods to extract the relevant information from the large amounts of industrial data that are produced by the complex processes. Statistical methods have been developed that can handle such large datasets and develop useful models. Common methods include principal component analysis (PCA) [16] and partial least squares (PLS) [17]. PCA is a powerful tool for data compression and information extraction that can simplify the model structure and improve the speed of operations. However, PCA can only deal with the correlations between vectors in the same matrix. To overcome this limitation, PLS was developed as an approach that models the correlation between independent variables and dependent variables. Since PLS only applies to linear systems or weakly nonlinear systems, many nonlinear PLS algorithms have been developed to handle nonlinear systems. The neural-network-based PLS algorithm [18] uses the nonlinear processing capability of a neural network to describe the relationship between variables. However, the determination of the network structure and the selection of network training algorithms are difficult problems. In addition, if there are too many datapoints, the model structure will be very complex and the accuracy will be difficult to guarantee.

On the other hand, considering that data-driven modeling methods use historical data for training, this raises the question of how to handle missing data. Along with issues such as the reliability of sensors and multirate sampling, missing data is common in practical industry process [19,20]. For example, in the aluminum electrolysis process, the alumina concentration is usually obtained manually by laboratory staff. Considering human factors and chemical examination equipment reliability, data loss occurs from time to time. In this case, this type of measurement has different effects on the soft sensor modeling process and state estimation performance. Therefore, in order to make the soft sensor more suitable for practical, complex industrial processes, the missing data problem needs to be taken seriously. Compared with the direct deletion of missing data, the data interpolation method [21] is better able to restore the real situation. Currently, data interpolation methods include the mean substitution method, the regression interpolation method, and the expectation maximization (EM) algorithm. Of these, the mean substitution method can cause biased estimates, and the regression interpolation method is built based on a complete data set, where the linear relationship between the variables with missing values and other variables is necessary, which, in many cases, cannot be satisfied. In fact, the EM algorithm has good practical value as an iterative algorithm for simplifying the maximum likelihood estimation when dealing with missing data in sample sets [22].

Recently, in order to evaluate the accuracy of the model output, the coefficient of determination approach has been considered. The coefficient of determination is the measurement of how well the regression model fits the data [23]. Feng [24] introduced the coefficient of determination as a criterion for comparing the best-wavelength partial least squares regression (PLSR) model with the full-wavelength model. Boyaci [25] used the coefficient of determination to evaluate the adulteration rate of coffee beans, thus ensuring coffee quality. However, these applications only consider the coefficient of determination as an evaluation index without applying it for the modeling process. In general, the coefficient of determination is a criterion that can evaluate the quality of a model and has a concise structure, so it is appropriate to apply it to the soft sensor development process to establish a simpler and more accurate model for complex industrial process.

Therefore, this paper develops a KPI-based soft sensor model with simple structure and high accuracy, using the coefficient of determination method, which also solves the missing data issue using the EM algorithm.

## 2. Background

### 2.1. The Gaussian Mixture Model

As a flexible and efficient tool for probabilistic data models, a Gaussian mixture model (GMM) can be used to define any complex probability distribution function and is, therefore widely used in many statistical data modelling applications. In this paper, GMM is used to approximate the joint probability distribution in the soft sensor probability model. The reason for introducing GMM is that, theoretically, any probability distribution can be approximated using the joint weighted Gaussian distribution [26]. 

If *x* represents a multidimensional random variable, then the joint probability distribution of the GMM is expressed as

(1) $$p\left(x|\Theta \right)=\sum_{l=1}^{M}\alpha_{l}p_{l}\left(x|\theta_{l}\right)\;$$

where *α_l_* is the mixing coefficient, which represents the prior probability of each mixed component; *M* is the number of mixed components; and $\sum_{l=1}^{M}\alpha_{l}=1$. $\Theta =\left(\theta_{1},\theta_{2},\cdots ,\theta_{M}\right)$ is the parameter vector of each mixed component, and each Gaussian probability density function *p_l_*(*x*) is determined by the parameter θ*_l_* = (μ*_l_*, Σ*_l_*), where μ*_l_* is the mean and Σ*_l_* is the covariance matrix. The GMM parameters α*_l_*, μ*_l_*, and Σ*_l_* (*l* = 1, 2, …, *M*) are estimated using the EM algorithm.

### 2.2. The Expectation Maximization Algorithm

The EM algorithm is a maximum likelihood estimation method for solving model distribution parameters from “incomplete data” and was first introduced in [27]. Each iteration of the algorithm involves two steps, called the expectation step (E-step) and the maximization step (M-step).

#### 2.2.1. E-Step

Given the observation data set *X* and the current parameters Γ^(*i*)^, the expectation of the log-likelihood function is called the *Q*-function which can be written as

(2) $$Q(\Gamma ,\Gamma^{\left(i\right)})=E\left[\log p\left(X,|\Gamma \right)|X,\Gamma^{\left(i\right)}\right]\;$$

where *γ* can represent missing data due to observational conditions and other reasons, and can also refer to hidden variables. Since the direct optimization of the likelihood function is usually very difficult, the relationship between *X*, Γ, and *γ* can be established by introducing an additional variable *γ* to achieve the purpose of simplifying the likelihood function.

#### 2.2.2. M-Step

A new parameter Γ^(*i+*1)^ is calculated by maximizing *Q*(Γ, Γ^(*i*)^) which was obtained from the E-step; that is,

(3) $$\Gamma^{\left(i+1\right)}=\underset{\Gamma }{\arg\max}Q(\Gamma ,\Gamma^{\left(i\right)})\;.\;$$

The iteration between the E- and M-steps continues until the elements of Γ are less than a given value.

### 2.3. The Coefficient of Determination

Analysis of variance is an approach for determining the significance and validity of a regression model using variances obtained from the data and model. The coefficient of determination is an analysis of variance approach that seeks to decompose the total variability in the data into various orthogonal components that can then be independently analyzed [23]. For the purposes of analyzing the regression, let the total sum of squares, denoted by TSS, be defined as

(4) $$TSS=\sum_{i=1}^{n}{\left(y_{i}-\bar{y}\right)}^{2}\;$$

where the real data set is represented as *y* = <*y*_1_, *y*_2_, …, *y_n_*> and $\bar{y}$ refers to the average of *y_i_*. Let the sum of squares due to regression, SSR, be defined as

(5) $$SSR=\sum_{i=1}^{n}{\left({\hat{y}}_{i}-\bar{y}\right)}^{2}\;$$

where ${\hat{y}}_{i}$ denotes the predicted value of the regression model for *y_i_*. The coefficient of determination *R*^2^ represents the ratio of SSR to TSS, that is,

(6) $$R^{2}=\frac{SSR}{TSS}\;.\;$$

Let the sum of squares due to the error, SSE, be defined as

(7) $$SSE=\sum_{i=1}^{n}{\left(y_{i}-{\hat{y}}_{i}\right)}^{2}.\;$$

It can be proved that TSS = SSR + SSE [23,28], so *R*^2^ can also be expressed as

(8) $$R^{2}=1-\frac{SSE}{TSS}=1-\frac{\sum_{i=1}^{n}{\left(y_{i}-{\hat{y}}_{i}\right)}^{2}}{\sum_{i=1}^{n}{\left(y_{i}-\bar{y}\right)}^{2}}\;.\;$$

## 3. Development of the Probabilistic Soft Sensor Model

**Figure 1:**
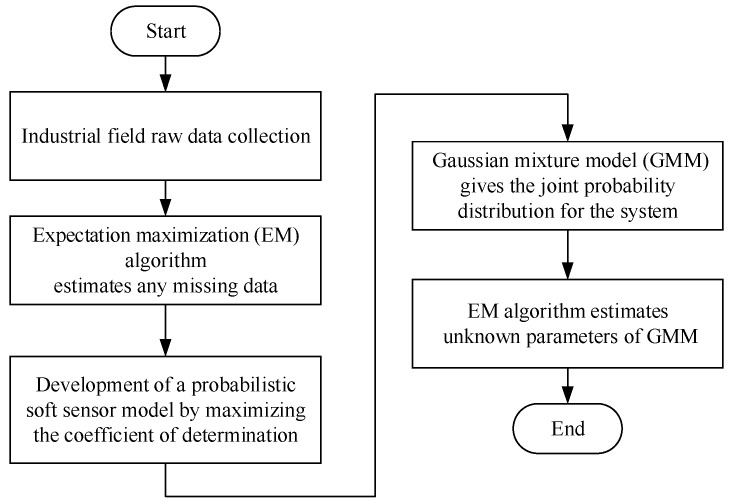
The flow chart of soft sensor development process.

In this section, in order to obtain more accurate KPI estimates, a soft sensor development approach based on maximizing the coefficient of determination is proposed. In addition, the problem of missing data in the training sample set is also considered. In order to more clearly describe the soft sensor development process, Figure 1 shows the modeling flow chart.

### 3.1. EM Algorithm Handing Missing Data

Let *X*_1_, *X*_2_, …, *X_n_* be a random sample from a *p*-variate normal population, where *X_j_* = (*x_j_*_1_, *x_j_*_2_, …, *x_j_*_p_), 1 ≤ *j* ≤ *n*, so the training sample set *X* can be written as

(9) $$X=\left(\begin{array}{l} X_{1}\\ X_{2}\\ \;\vdots \\ X_{n} \end{array}\right)=\left[\begin{array}{llll} x_{11}, & x_{12}, & \cdots , & x_{1p}\\ x_{21}, & x_{22}, & \cdots , & x_{2p}\\ \vdots & & \ddots & \vdots \\ x_{n1}, & x_{n2}, & \cdots , & x_{np} \end{array}\right]\;.\;$$

The basic steps for processing missing data using the EM algorithm are given in [29].

#### 3.1.1. E-Step: Prediction

For each sample *X_j_* containing missing values, *X_j_* = (*m_j_*, *a_j_*), where *m_j_* is the missing value and *a_j_* is the available values. Given the population mean and variance, ${\tilde{\mu }}^{i}$ and ${\tilde{\Sigma }}^{i}$, from the *i*th iteration and *a_j_*, we use the expectation of the conditional normal distribution of *m_j_* as the estimate of the missing value. The (*i* + 1)th iteration is

(10) $${\tilde{m}}_{j}^{i+1}=E\left(m_{j}|a_{j},{\tilde{\mu }}^{i},{\tilde{\sum }}^{i}\right)={\tilde{\mu }}_{m}^{i}+{\tilde{\sum }}_{ma}^{i}{\left({\tilde{\sum }}_{aa}^{i}\right)}^{-1}\left(a_{j}-{\tilde{\mu }}_{a}^{i}\right)\;$$

where ${\tilde{\mu }}^{i}$ is a *p* × 1 matrix defined as ${\tilde{\mu }}^{i}={\left[{\tilde{\mu }}_{m}^{i},{\tilde{\mu }}_{a}^{i}\right]}^{\prime }$, ${\tilde{\mu }}_{m}^{i}$ is the mean of the missing part, and ${\tilde{\mu }}_{a}^{i}$ is the mean of the available part. In addition, ${\tilde{\Sigma }}^{i}$ can be written as

(11) $${\tilde{\sum }}^{i}=\left[\begin{array}{l} {\tilde{\sum }}_{mm}^{i}{\tilde{\sum }}_{ma}^{i}\\ {\tilde{\sum }}_{am}^{i}{\tilde{\sum }}_{aa}^{i} \end{array}\right]\;.\;$$

#### 3.1.2. M-Step: Estimation

We compute the maximum likelihood estimates as follows:

(12) $${\tilde{\mu }}^{i+1}={\bar{X}}^{i+1}\;$$

(13) $${\tilde{\sum }}^{i+1}=\frac{\left(n-1\right)S^{i+1}}{n}\;$$

where ${\bar{X}}^{i+1}$ is the mean of the samples and *S^i+^*^1^ is the sample standard deviation, and they are all sufficient statistics. For a normal population, the importance of sufficient statistics is that the total information about *μ* and Σ in the data matrix *X* is contained in $\bar{X}$ and *S*, regardless of the sample size *n*. By transforming $\bar{X}$ and *S*, two new sufficient statistics *T*_1_ and *T*_2_ [29], given by

(14) $$T_{1}=n\bar{X}\;$$

(15) $$T_{2}=\left(n-1\right)S+n\bar{X}{\bar{X}}^{\prime }\;$$

are obtained. Combining Equations (14) and (15) with Equations (12) and (13) gives

(16) $${\tilde{\mu }}^{i+1}=\frac{T_{1}^{i+1}}{n}\;$$

(17) $${\tilde{\sum }}^{i+1}=\frac{1}{n}T_{2}{}^{i+1}-{\tilde{\mu }}^{i+1}{\left({\tilde{\mu }}^{i+1}\right)}^{\prime }\;$$

where

(18) $${\tilde{m_{j}m_{j}{}^{\prime }}}^{i+1}=E\left(m_{j}m_{j}{}^{\prime }|a_{j},{\tilde{\mu }}^{i},{\tilde{\sum }}^{i}\right)={\tilde{\sum }}^{i}{}_{mm}-{\tilde{\sum }}^{i}{}_{ma}{\left({\tilde{\sum }}^{i}{}_{aa}\right)}^{-1}{\tilde{\sum }}^{i}{}_{am}+{\tilde{m}}_{j}^{i+1}{\left({\tilde{m}}_{j}^{i+1}\right)}^{\prime }\;$$

(19) $${\tilde{m_{j}^{}a_{j}{}^{\prime }}}^{i+1}=E\left(m_{j}^{}a_{j}{}^{\prime }|a_{j},{\tilde{\mu }}^{i},{\tilde{\sum }}^{i}\right)={\tilde{m}}_{j}^{i+1}{\left(a_{j}^{}\right)}^{\prime }.\;$$

The iteration between the E- and M-steps continues until the elements of $\tilde{\mu }$ and $\tilde{\Sigma }$ are less than a given value. Therefore, the iteration result $\tilde{m}$ is the optimal substitution for the missing values, resulting in a complete training sample set *X*.

### 3.2. Soft Sensor Development Approach Based on the Coefficient of Determination Maximization Strategy

For the complete training sample set *X* obtained from Section 3.1, which can be written as

(20) $$X=\left[\begin{array}{llll} x_{11}, & x_{12}, & \cdots , & x_{1p}\\ x_{21}, & x_{22}, & \cdots , & x_{2p}\\ \vdots & & \ddots & \vdots \\ x_{n1}, & x_{n2}, & \cdots , & x_{np} \end{array}\right]\;$$

let $\left(x_{1},x_{2},\cdots x_{p-1}\right)$ denote the secondary variables, and *x_p_* denote the KPI. Our objective is to estimate *x_p_* from $\left(x_{1},x_{2},\cdots x_{p-1}\right)$.

*R*^2^ measures the fraction of the total variance in the model explained by the regression with the given variables [23]. The range of *R*^2^ is [0,1]. Let *x_p_* be the *y* mentioned in Section 2.3. Then, the coefficient of determination is

(21) $$R^{2}=1-\frac{\sum_{i=1}^{n}{\left(x_{ip}-{\hat{x}}_{ip}\right)}^{2}}{\sum_{i=1}^{n}{\left(x_{ip}-{\bar{x}}_{p}\right)}^{2}}\;.\;$$

If the secondary variables in the soft sensor model do not account for the variance of *x_p_*, the estimate of *x_ip_*, denoted ${\hat{x}}_{ip}$, is exactly equal to the sample mean of *x_ip_*, denoted ${\bar{x}}_{ip}$. In this case, SSR is 0 and SSE equal to TSS, so *R*^2^ = 0. On the other hand, if $\left(x_{i1},x_{i2},\cdots x_{i(p-1)}\right)$ fully explains the variance of *x_ip_*, for *i* = 1, 2,…, *n*, it follows that *x_ip_* = ${\bar{x}}_{ip}$, i.e., each error is zero and SSR = TSS, so *R*^2^ = 1. In general, *R*^2^ does not take the extreme values 0 or 1, but instead takes a certain value between the two [28]. For the case where the number of variables, *p*, is much smaller than the sample number *n*, the closer *R*^2^ is to 1, the better the model. Therefore, when the model for the KPI maximizes *R*^2^, it becomes the best estimate of the KPI, that is,

(22) $$1-\frac{\sum_{i=1}^{n}{\left(x_{ip}-{\tilde{x}}_{ip}\right)}^{2}}{\sum_{i=1}^{n}{\left(x_{ip}-{\bar{x}}_{p}\right)}^{2}}=\max\left[1-\frac{\sum_{i=1}^{n}{\left(x_{ip}-K_{i}\right)}^{2}}{\sum_{i=1}^{n}{\left(x_{ip}-{\bar{x}}_{p}\right)}^{2}}\right]\;$$

where ${\tilde{x}}_{ip}$ is the best estimate of *x_ip_*, and *K_i_* represents all possible estimates of *x_ip_*. Simplifying the above equation gives

(23) $$\frac{\sum_{i=1}^{n}{\left(x_{ip}-{\tilde{x}}_{ip}\right)}^{2}}{\sum_{i=1}^{n}{\left(x_{ip}-{\bar{x}}_{p}\right)}^{2}}=\min\left[\frac{\sum_{i=1}^{n}{\left(x_{ip}-K_{i}\right)}^{2}}{\sum_{i=1}^{n}{\left(x_{ip}-{\bar{x}}_{p}\right)}^{2}}\right]\;$$

where *x_ip_* and ${\bar{x}}_{p}$ are both computed values. Equation (23) can then be written as

(24) $$\sum_{i=1}^{n}{\left(x_{ip}-{\tilde{x}}_{ip}\right)}^{2}=\min\left[\sum_{i=1}^{n}{\left(x_{ip}-K_{i}\right)}^{2}\right]\;.\;$$

Multiplying Equation (24) on both sides by *n*^−1^ gives

(25) $$\frac{1}{n}\sum_{i=1}^{n}{\left(x_{ip}-{\tilde{x}}_{ip}\right)}^{2}=\min\left[\frac{1}{n}\sum_{i=1}^{n}{\left(x_{ip}-K_{i}\right)}^{2}\right]\;.\;$$

Considering that the mathematical expectation of a discrete random variable is

(26) $$E\left(x\right)=\sum_{i}x_{i}p_{i}\;$$

where *x_i_* represents the *i*th value of the random variable *x* and *p_i_* represents its probability, Equation (26) can be expressed as

(27) $$E\left\{{\left\|x_{p}-{\tilde{x}}_{p}\right\|}^{2}\right\}=\min\;E\left\{{\left\|x_{p}-K\right\|}^{2}\right\}\;$$

where *K* denotes all possible estimates of the KPI *x_p_*, and ${\tilde{x}}_{p}$ represents the best estimate of the KPI when the coefficient of determination *R*^2^ is maximized. Since *x_p_* is derived from the soft sensor models and secondary variables, the above equation can be written as

(28) $${\tilde{x}}_{p}=\underset{K}{\arg\min}E\left[{\left\|x_{p}-K\right\|}^{2}|\left(x_{1},x_{2},\cdots x_{p-1}\right)\right]\;.\;$$

In order to establish a more direct connection between ${\tilde{x}}_{p}$ and (*x_i_*_1_, *x_i_*_2_, …, *x_i_*_(*p*–1)_), the left-hand side of Equation (28) will be simplified further. Firstly, it can be noted that *K* does not have an impact on the simplification, that is,

(29) $$\begin{array}{cc} & E\left[{\left\|x_{p}-K\right\|}^{2}|\left(x_{1},x_{2},\cdots x_{p-1}\right)\right]\\ = & E\left[{\left\|x_{p}-E\left(x_{p}|\left(x_{1},x_{2},\cdots x_{p-1}\right)\right)+E\left(x_{p}|\left(x_{1},x_{2},\cdots x_{p-1}\right)\right)-K\right\|}^{2}|\left(x_{1},x_{2},\cdots x_{p-1}\right)\right]\;\\ = & E\left[{\left\|x_{p}-E\left(x_{p}|\left(x_{1},x_{2},\cdots x_{p-1}\right)\right)\right\|}^{2}|\left(x_{1},x_{2},\cdots x_{p-1}\right)\right]+E\left[{\left\|E\left(x_{p}|\left(x_{1},x_{2},\cdots x_{p-1}\right)\right)-K\right\|}^{2}|\left(x_{1},x_{2},\cdots x_{p-1}\right)\right]\;\\ & +E\left[{\left[x_{p}-E\left(x_{p}|\left(x_{1},x_{2},\cdots x_{p-1}\right)\right)\right]}^{T}\left[E\left(x_{p}|\left(x_{1},x_{2},\cdots x_{p-1}\right)\right)-K\right]|\left(x_{1},x_{2},\cdots x_{p-1}\right)\right]\;\\ & +E\left[{\left[E\left(x_{p}|\left(x_{1},x_{2},\cdots x_{p-1}\right)\right)-K\right]}^{T}\left[x_{p}-E\left(x_{p}|\left(x_{1},x_{2},\cdots x_{p-1}\right)\right)\right]|\left(x_{1},x_{2},\cdots x_{p-1}\right)\right]\; \end{array}$$

In order to minimize the above equation, the following should hold:

(30) $$K=E\left[x_{p}|\left(x_{1},x_{2},\cdots x_{p-1}\right)\right]\;$$

which can be rewritten as

(31) $${\tilde{x}}_{p}=E\left[x_{p}|\left(x_{1},x_{2},\cdots x_{p-1}\right)\right]\;.\;$$

Furthermore, $E\left[x_{p}|\left(x_{1},x_{2},\cdots x_{p-1}\right)\right]$ can be expanded according to the definition of expectation, giving

(32) $$\begin{array}{cc} {\tilde{x}}_{p} & =E\left[x_{p}|\left(x_{1},x_{2},\cdots x_{p-1}\right)\right]\;\\ & =\int x_{p}p\left[x_{p}|\left(x_{1},x_{2},\cdots x_{p-1}\right)\right]dx_{p}\;.\\ & =\int x_{p}\frac{p\left(x_{1},x_{2},\cdots x_{p-1},x_{p}\right)}{p\left(x_{1},x_{2},\cdots x_{p-1}\right)}dx_{p} \end{array}$$

Thus, this establishes the basic framework of the probabilistic soft sensor model with KPI optimal estimation.

The next part is to solve the joint probability distribution in the model.

In this paper, GMM is used to approximate the joint probability distribution. Let $p\left(x_{e}\right)=p\left(x_{1},x_{2},\cdots x_{p-1}\right)$; that is,

(33) $$p\left(x_{e}\right)=\sum_{j=1}^{M}\alpha_{j}p\left(x_{je}|\theta_{j}\right)\;$$

(34) $$p\left(x_{e},x_{p}\right)=\sum_{l=1}^{M}\alpha_{l}p\left(x_{le},x_{lp}|\theta_{l}\right)\;.\;$$

In order to deduce the specific representation of the KPI optimal estimation ${\tilde{x}}_{p}$ under the proposed probabilistic soft sensor model, we first introduce Lemma 1.

**Lemma** **1.** [30] *Let G(x; μ, Σ) be a multidimensional normal density function with mean μ and covariance matrix Σ. Let* $x^{T}=\left(x_{1}^{T},x_{2}^{T}\right)$*,* $\mu^{T}=\left(\mu_{1}^{T},\mu_{2}^{T}\right)$*, and* $\Sigma =\left[\begin{array}{l} \Sigma_{11}\Sigma_{12}\\ \Sigma_{21}\Sigma_{22} \end{array}\right]$*; then, the joint probability density is*

(35) $$p\left(x\right)=G\left(x_{1};\mu_{1},\Sigma_{11}\right)G\left(x_{2};\mu_{x_{2}|x_{1}},\Sigma_{x_{2}|x_{1}}\right)\;$$

*where*

(36) $$\mu_{x_{2}|x_{1}}=\mu_{2}-\Sigma_{21}\Sigma_{11}^{-1}\left(\mu_{1}-x_{1}\right)\;$$

(37) $$\Sigma_{x_{2}|x_{1}}=\Sigma_{22}-\Sigma_{21}\Sigma_{11}^{-1}\Sigma_{12}\;.\;$$

**Proof.**  The details of the proof can be found in [30].

Using Lemma 1, it follows that

(38) $$\begin{array}{cc} & p\left(x_{le},x_{lp}\right)\\ = & G\left(x_{l};\mu_{l},\Sigma_{l}\right)\\ = & G\left(x_{le};\mu_{le},\Sigma_{lee}\right)G\left(x_{lp};\mu_{lp|e},\Sigma_{lp|e}\right) \end{array}$$

where $\mu_{l}=\left(\mu_{le}^{T},\mu_{lp}^{T}\right)$ and $\Sigma_{l}=\left[\begin{array}{l} \Sigma_{lee}\Sigma_{lep}\\ \Sigma_{lpe}\Sigma_{lpp} \end{array}\right]$. Therefore, Equations (33) and (34) can be written as

(39) $$p\left(x_{e}\right)=\sum_{j=1}^{M}\alpha_{j}G\left(x_{je};\mu_{je},\Sigma_{jee}\right)\;$$

(40) $$p\left(x_{e},x_{p}\right)=\sum_{l=1}^{M}\alpha_{l}G\left(x_{le};\mu_{le},\Sigma_{lee}\right)G\left(x_{lp};\mu_{lp|e},\Sigma_{lp|e}\right)\;.\;$$

Substituting Equations (39) and (40) into Equation (32) gives

(41) $$\begin{array}{cc} {\tilde{x}}_{p} & =\int x_{p}\frac{p\left(x_{e},x_{p}\right)}{p\left(x_{e}\right)}dx_{p}\\ & =\int x_{p}\frac{\sum_{l=1}^{M}\alpha_{l}G\left(x_{le};\mu_{le},\Sigma_{lee}\right)G\left(x_{lp};\mu_{lp|e},\Sigma_{lp|e}\right)}{\sum_{j=1}^{M}\alpha_{j}G\left(x_{je};\mu_{je},\Sigma_{jee}\right)}dx_{p}\;. \end{array}$$

Extracting the sum in the numerator to outside the integral gives

(42) $${\tilde{x}}_{p}=\sum_{l=1}^{M}\int x_{p}\frac{\alpha_{l}G\left(x_{le};\mu_{le},\Sigma_{lee}\right)G\left(x_{lp};\mu_{lp|e},\Sigma_{lp|e}\right)}{\sum_{j=1}^{M}\alpha_{j}G\left(x_{je};\mu_{je},\Sigma_{jee}\right)}dx_{p}\;.\;$$

In order to make the derivation more concise, the positions of some factors in the integral are changed as follows:

(43) $$\begin{array}{cc} {\tilde{x}}_{p} & =\sum_{l=1}^{M}\int \frac{\alpha_{l}G\left(x_{le};\mu_{le},\Sigma_{lee}\right)}{\sum_{j=1}^{M}\alpha_{j}G\left(x_{je};\mu_{je},\Sigma_{jee}\right)}x_{p}G\left(x_{lp};\mu_{lp|e},\Sigma_{lp|e}\right)dx_{p}\\ & =\sum_{l=1}^{M}\frac{\alpha_{l}G\left(x_{le};\mu_{le},\Sigma_{lee}\right)}{\sum_{j=1}^{M}\alpha_{j}G\left(x_{je};\mu_{je},\Sigma_{jee}\right)}\int x_{p}G\left(x_{lp};\mu_{lp|e},\Sigma_{lp|e}\right)dx_{p}\;. \end{array}$$

When the integral part is the conditional expectation, the above equation can be simplified to

(44) $$\;{\tilde{x}}_{p}\;=\sum_{l=1}^{M}\frac{\alpha_{l}G\left(x_{le};\mu_{le},\Sigma_{lee}\right)}{\sum_{j=1}^{M}\alpha_{j}G\left(x_{je};\mu_{je},\Sigma_{jee}\right)}\mu_{lp|e}\;.\;$$

Therefore, the detailed soft sensor model expression of the KPI optimal estimation is obtained.

In this paper, unknown parameters in the model are estimated using the EM algorithm. The iterative equations of the EM algorithm for estimating the GMM parameters are [31]

(45) $$\mu_{l}^{\left(i+1\right)}=\frac{\sum_{j=1}^{n}\gamma_{jl}^{\left(i+1\right)}X_{j}}{\sum_{j=1}^{n}\gamma_{jl}^{\left(i+1\right)}},\;\Sigma_{l}^{\left(i+1\right)}=\frac{\sum_{j=1}^{n}\gamma_{jl}^{\left(i+1\right)}{\left(X_{j}-\mu^{\left(i\right)}\right)}^{2}}{\sum_{j=1}^{n}\gamma_{jl}^{\left(i+1\right)}},\;\alpha_{l}^{\left(i+1\right)}=\frac{\sum_{j=1}^{n}\gamma_{jl}^{\left(i+1\right)}}{n}\;$$

where *γ_jl_* represents the responsivity of the mixed component *l* on the training sample data *X_j_*. It can be written as

(46) $$\gamma_{jl}^{\left(i+1\right)}=\frac{\alpha_{l}p\left(X_{j}|\theta_{l}\right)}{\sum_{l=1}^{M}\alpha_{l}p\left(X_{j}|\theta_{l}\right)}\;.\;$$

Consequently, the above steps give the GMM parameters, and the KPI optimal estimate ${\tilde{x}}_{p}$ follows.

## 4. Case Study

In this section, the effectiveness and feasibility of the proposed soft sensor model approach based on maximizing the coefficient of determination are evaluated through an industrial aluminum electrolytic production process. To show the advantages of the probabilistic soft sensor framework, the estimations are compared with the real values. For performance evaluation, the root-mean-squared error (RMSE) index is used.

### 4.1. Soft Sensor Development for Industrial Aluminum Electrolytic Process

**Figure 2:**
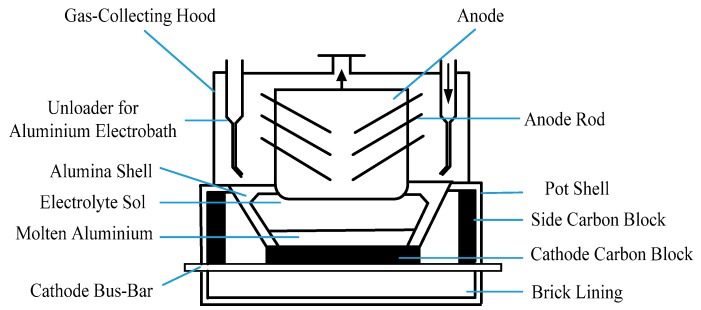
The internal structure of the aluminum electrolytic cell.

Aluminum is widely used in construction and electrical industries [32]. The main method currently chosen for smelting aluminum plants is the cryolite–alumina molten salt electrolysis process, in which the electrochemical reaction process takes place in an electrolytic cell. Figure 2 shows the internal structure of the electrolytic cell.

Molten cryolite is a solvent in which aluminum oxide is dissolved as a solute, forming a melt with good electrical conductivity. Carbon materials are used as cathodes and anodes, and a direct current is passed through them. The thermal energy of the direct current is used to melt the cryolite and maintain a constant electrolysis temperature. Furthermore, the electrochemical reaction occurs between the two electrodes, where the product at the cathode is aluminum liquid, and carbon dioxide and other gases are generated at the anode. The chemical reaction of the electrolytic process is

2Al_2_O_3_ + 3C → 4Al + 3CO_2__↑_.

**Figure 3:**
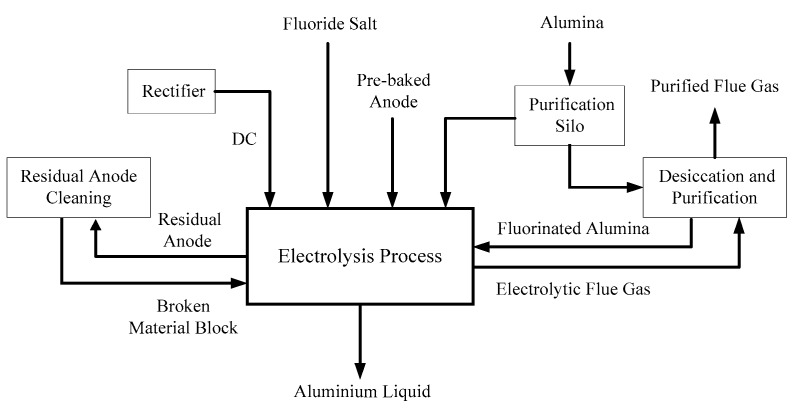
The process flow diagram of the aluminum electrolysis process.

The chemical reaction can produce gases other than carbon dioxide and carbon monoxide, as well as fluorocarbon gases. The gas purifying device uses alumina and fluorine generated in the mixed gas to produce fluorinated alumina, and the fluorinated alumina is then recycled to the electrolytic cell for chemical reaction. Figure 3 shows the process flow diagram of the aluminum electrolysis process.

The main control goal of the aluminum electrolysis process is to keep the alumina concentration in the electrolysis cell stable within a certain range, preferably between 1.5% and 3.5% [33]. The control of alumina concentration relates to energy consumption and economic benefits of the aluminum electrolytic production process. On one hand, when the alumina concentration is too low, an additional chemical reaction occurs at the anode, which can easily cause a sudden rise in the cell voltage and the energy balance of the cell is destroyed. On the other hand, when the concentration reaches saturation, if the feeder continues to add alumina at the time, the raw material will be deposited at the bottom of the cell, so that the resistance increases and the current efficiency becomes low. Therefore, it is necessary to keep the alumina concentration in the proper range. 

**Figure 4:**
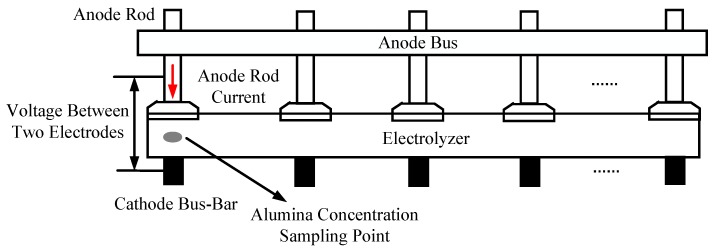
Schematic diagram of the variable collection system.

In soft sensor development for the aluminum electrolytic process, the measurable variables, the voltage *x*_1_ between the two electrodes obtained by the first voltage measuring instrument; the anode conductor current *x*_2_; the voltage *x*_3_ between the two electrodes obtained by the second voltage measuring instrument; and the alumina concentration *x*_4_ provided by an electrochemical analyzer, were selected as the secondary variables. The interelectrode voltage refers to the voltage between the anode guide and the corresponding cathode steel bar. The alumina concentration *y* provided by the laboratory is the primary variable for the model. Figure 4 shows a diagram of the process measurement system.

The variables *x*_1_(*k*), *x*_2_(*k*), *x*_3_(*k*), *x*_4_(*k*), and *y*(*k*) form the joint probability distribution

(47) $$p\left(x(k)\right)=p(x_{1}(k),x_{2}(k),x_{3}(k),x_{4}(k),y(k))\;.\;$$

The soft sensor was then developed according to the process described in Section 3 of this paper. It is assumed that *M* = 2.

### 4.2. Experimental Results

#### 4.2.1. EM Algorithm and Missing Values

We took 600 complete data groups from the training sample set, and deleted 10%, 20%, or 30% of the alumina concentration variable data. Then, the mean substitution method, the regression interpolation method, and the EM algorithm were used to process the sample set with missing values. Table 1, Table 2 and Table 3 show the mean and RMSE of the alumina concentration sample set for the three method simulations for missing ratios of 10%, 20%, and 30%.

First, comparing the mean value, we can see from the above tables that the means of the regression interpolation method and the EM data interpolation method are closer to the mean of the real value set, and the mean substitution method is less effective. Obviously, the RMSE of the EM data interpolation method is much smaller than that of the regression interpolation method. Therefore, the accuracy and effectiveness of the EM data interpolation method in processing missing values is verified. Further, if there is a problem with missing values in the practical industrial process, the EM algorithm can be selected for data interpolation.

#### 4.2.2. Experimental Results of the Soft Sensor Model Based on Maximizing the Coefficient of Determination

**Figure 5:**
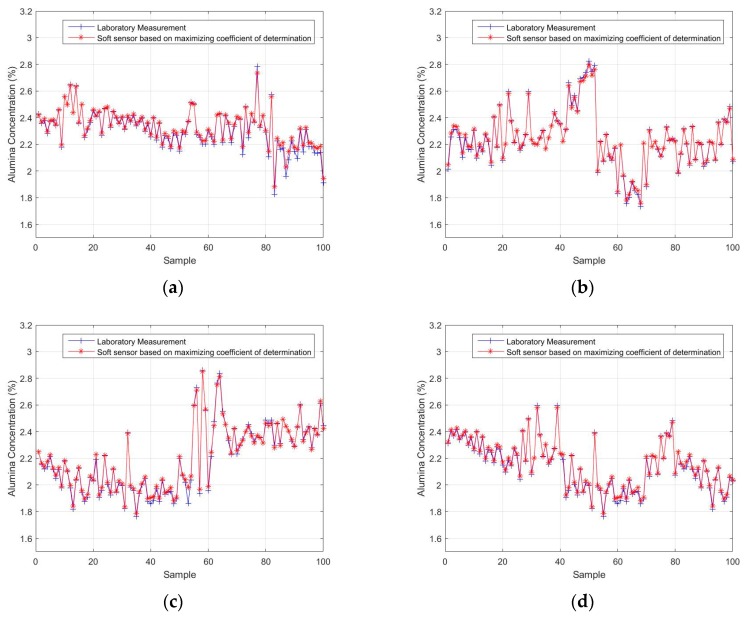
The soft-sensor-estimated alumina concentrations, based on maximizing the coefficient of determination, compared with the actual laboratory measurement using (**a**) the first test subset, (**b**) the second test subset, (**c**) the third test subset, and (**d**) the fourth test subset.

In order to verify the feasibility of the proposed approach, a test sample set was used to validate the designed soft sensor model. The test sample set was divided into four subsets of 100 samples. The actual alumina concentration measurement obtained from the laboratory was compared with the output of the soft sensor model to acquire an estimated performance evaluation of the model. The results are shown in Figure 5. Figure 5a–d show the estimated alumina concentrations based on the first, second, third, and fourth test subsets, respectively. Table 4 shows the root-mean-square errors (RMSE) of the four test subsets. It can be seen that, overall, the soft sensor model based on maximizing the coefficient of determination can accurately track the overall trends in the process. The alumina concentration output by the model is approximately the same as the actual laboratory measurement.

#### 4.2.3. Comparison with BP and LSSVM

**Figure 6:**
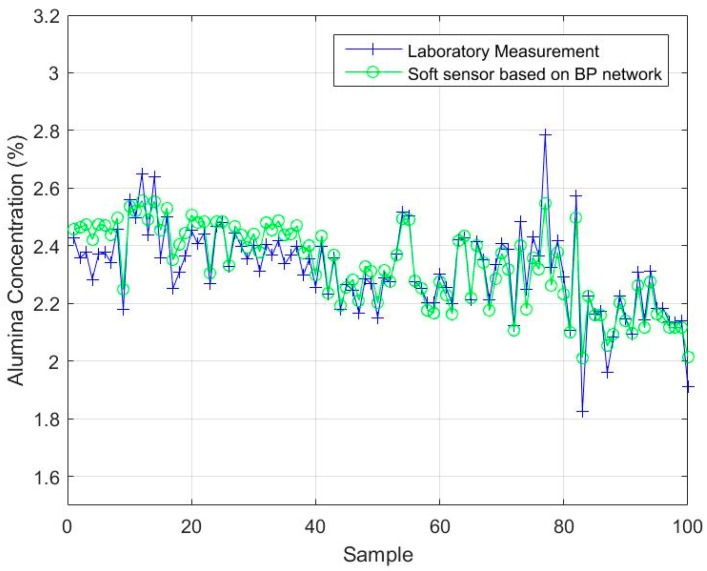
The estimated values of the soft sensor based on a backpropagation (BP) network compared with actual laboratory measurements.

**Figure 7:**
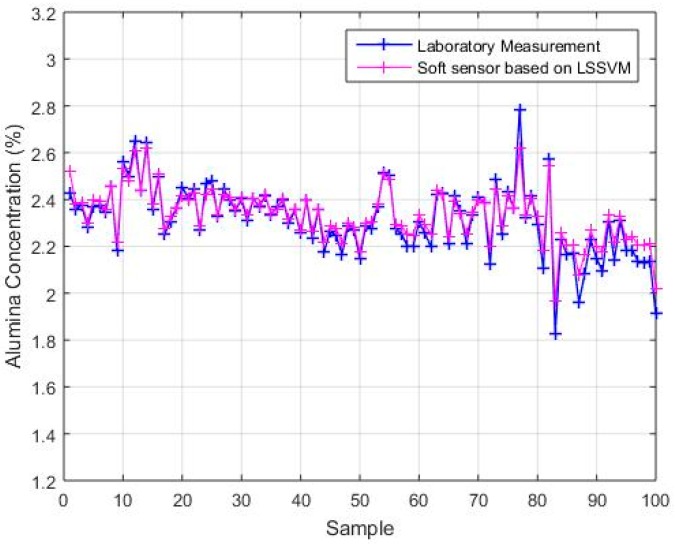
The estimated values of the soft sensor based on LSSVM compared with actual laboratory measurements.

The backpropagation (BP) neural network and the least-squares, support vector machine (LSSVM) model were applied to the test sample set, and the first test subset was used for performance comparison. The parameters of the comparison algorithms were determined as follows: The number of hidden layer nodes in the BP neural network model was 100 and the activation function of the hidden layer was a sigmoid [34]. The kernel function of the LSSVM model was the radial basis function (RBF), and the kernel parameter and regular parameter were 1 and 20, respectively [34]. For each model, the number of secondary variables was 4, and the number of primary variables was 1. It could be seen that the two comparison models need different parameters in order to achieve an accurate estimation performance, while this is not necessary for the soft sensor model based on maximizing the coefficient of determination. The estimated results are shown in Figure 6 and Figure 7. Figure 6 shows the estimated values of the soft sensor based on the BP neural network for the first test subset, and Figure 7 shows the estimated values of the soft sensor based on the LSSVM for the first test subset. It can be seen from Figure 6 that the soft sensor based on a BP neural network can roughly follow the trend of the laboratory measurements, but the error is still large at many points. It can be seen from Figure 7 that the overall performance of the soft sensor based on LSSVM is better than that based on a BP neural network, but compared with Figure 5a, it is obvious that the estimation of some extreme points is not as accurate as that given by the soft sensor based on maximizing the coefficient of determination.

**Figure 8:**
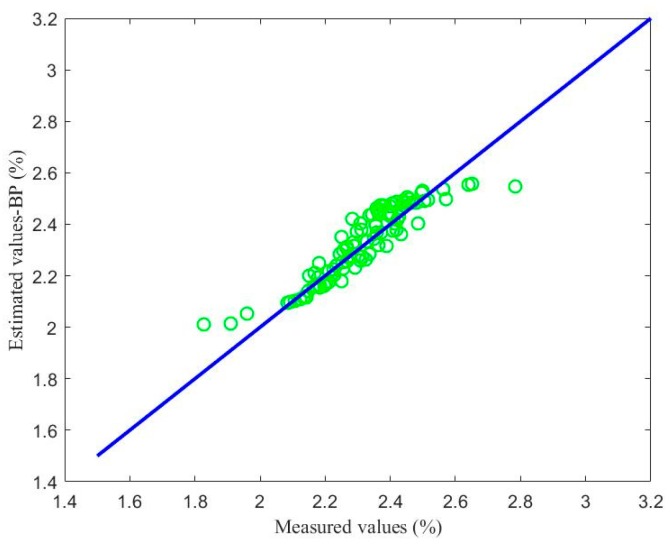
Comparison between the soft sensor based on a BP neural network and laboratory measurements.

**Figure 9:**
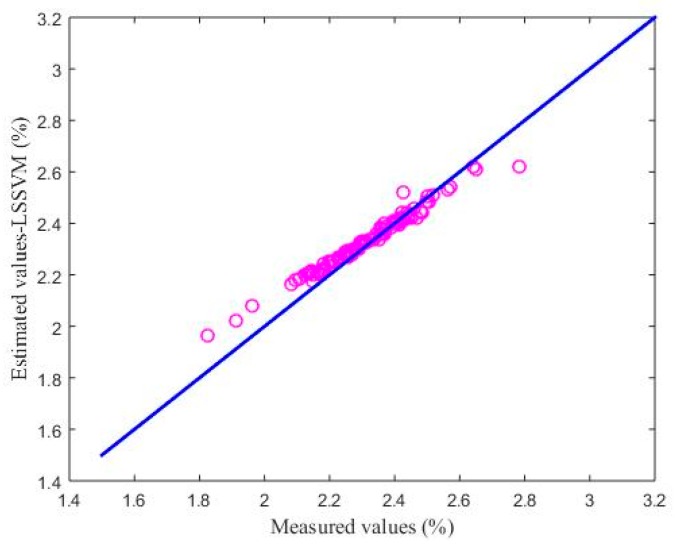
Comparison between the soft sensor based on LSSVM and laboratory measurements.

**Figure 10:**
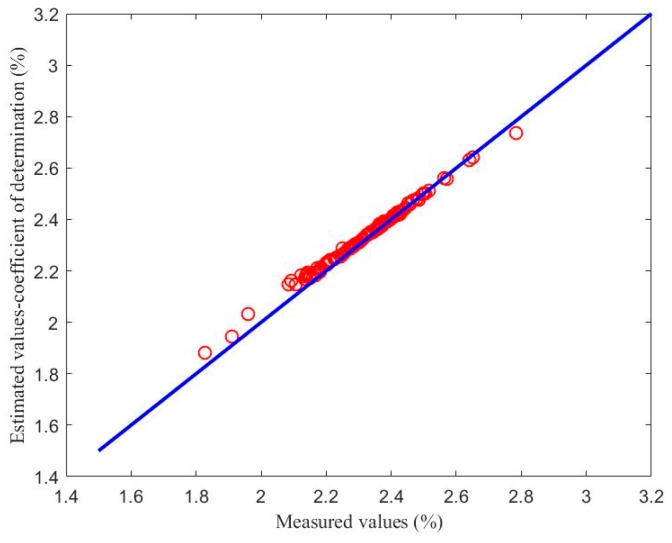
Comparison between the soft sensor based on maximizing the coefficient of determination and laboratory measurements.

Figure 8, Figure 9 and Figure 10 show the soft sensor estimates based on different modelling methods as a function of the laboratory measurements. The green circles show the BP neural network model; the purple circles the LSSVM model; and the red circles the proposed coefficient of determination maximization model. In the ideal case, the circles should lie on the blue *y* = *x* line. In practice, deviations from this behavior can provide information about the accuracy of the models. The BP neural network soft sensor produces a soft sensor system that has a consistent bias, since the values are consistently located above the *y* = *x* line. Furthermore, the bias in the LSSVM soft sensor model is smaller, but there also seems to be a calibration issue, since the data does not lie parallel to the *y* = *x* line. Finally, the proposed model has the smallest deviations and the most ideal performance.

To better illustrate the performance of the proposed soft sensor model, Table 5 shows the RMSE values for the different methods. As can be seen from Table 5, the RMSE of the proposed method is smallest, which means that the estimation effect of the proposed model is better than those of the BP neural network model and the LSSVM model.

## 5. Conclusions

In this paper, a new KPI estimation method for probabilistic soft sensor development is proposed based on maximizing the coefficient of determination. The joint probability distribution in the probability model is approximated using GMM, while the EM algorithm is used to estimate the GMM parameters. In addition to providing accurate, real-time estimates of the KPIs, this paper also considers the missing values that training sample sets often face and uses the EM algorithm for processing. The resulting soft sensor design method was tested on a case study of the alumina extraction process, which shows that the proposed method can provide alumina concentration estimations that are consistent with the actual measurements obtained from laboratory tests. Future work will focus on applying the proposed soft sensor development approach to solving various problems such as dealing with dynamic, non-Gaussian, or batch processes.

## Figures and Tables

**Table 1:** Comparison of three data interpolation methods for a 10% missing rate.

	Mean Substitution Method	Regression Interpolation Method	EM Algorithm	Real Value
Mean	2.4133	2.4225	2.4225	2.4259
RMSE	0.0867	0.4209	0.0698	0

**Table 2:** Comparison of three data interpolation methods for a 20% missing rate.

	Mean Substitution Method	Regression Interpolation Method	EM Algorithm	Real Value
Mean	2.4139	2.4217	2.4215	2.4259
RMSE	0.1451	0.4075	0.1361	0

**Table 3:** Comparison of three data interpolation methods for a 30% missing rate.

	Mean Substitution Method	Regression Interpolation Method	EM Algorithm	Real Value
Mean	2.4140	2.4204	2.41198	2.4259
RMSE	0.1700	0.4068		0

**Table 4:** The RMSE values of the four test subsets.

Test Subset	RMSE
First	0.0231
Second	0.0145
Third	0.0209
Fourth	0.0155

**Table 5:** The comparison of the RMSE between the three modelling methods.

Method	RMSE
BP neural network	0.0616
LSSVM	0.0431
Maximizing the Coefficient of Determination	0.0231
